# Medical and Philosophical Intelligence

**Published:** 1811-09

**Authors:** 


### MEDICAL AND PHILOSOPHICAL INTELLIGENCE.

ROYAL SOCIETY, July 4.--A paper by Dr. Wells was read on vision. The purport of the Author's observations was, that the focal distance of the eye depends chiefly on the contractability of the muscles, and that the latter is much greater in youth than in advanced age. In youth the eye is capable of accommodating itself to the light and the distance of external objects, but in old age this contractile power of the muscles ceases, and the focal distance of the eye becomes shorter, and more fixed to a determinate point. From experiments made with the Atropos Belladonna, it appeared that this plant increased the action of the ocular muscles in the young, but not in the old subject. From these observations it is inferred, that short sight is less owing to the prominence of the eye, than to the flexibility of the muscles which direct it.

---

From the following Circular, it appears that a Medical School has lately been established at Boston, New England ; where, also, it is in contemplation to build a new hospital, and the State has agreed to contribute 40,000 dollars towards it.

*Boston.*

" Sir,—The medical institution which has heretofore existed in Cambridge, has lately undergone such important alterations, that we have presumed some information of its actual arrangements might be agreeable to you and useful to some of your friends.

" Every physician in New England has, no doubt, been aware of the difficulty in obtaining a good medical education in this part of the United States. This difficulty has arisen principally from two sources. The first was the want of long and minute courses of lectures. The second, the deficiency of opportunities for exhibiting to students actual cases of disease ; and the practice employed for them, in medicine and surgery.

We expect to be able to remove these embarrassments in future. The Honourable and Reverend Corporation and Board of Overseers of Harvard College have established a medical school, in the town of Boston. The professors, all of whom except one reside there, will be able to devote a longer time to the lectures, than they formerly did, and thus to render them more minute and more instructive. The second source of difficulty has been obviated by the liberality of the Honourable Board of Overseers of the poor of the town of Boston, who have committed the charge of the hospital department of the Alms House to such of the medical professors of the university, as have been recommended by the Corporation. This department contains about fifty patients, afflicted with a variety of diseases, which are the objects of both medicine and surgery. Operations in surgery also occasionally present themselves. These will afford very important practical advantages, all which will be accessible to the medical students.

" The

"The following courses of lectures will be commenced in Boston, on the first Wednesday in December.

"Anatomy and Physiology, Surgery and Midwifery, by Dr. Warren, Sen: and Dr. Warren, Jun.

"Theory and Practice of Physic, by Dr. Waterhouse.

"Chemistry and Materia Medica, by Dr. Dexter, and Dr. Gorham.

"Clinical Medicine, by Dr. Jackson.

"The number of lectures will probably be about fifty, certainly not more than sixty, during the present season. This number will be gradually increased till it equal that given in the most respectable seminaries in the United States. The lectures will be delivered daily.

"The object of the new Professorship of clinical medicine is, 'to point out at the bedside of such sick persons, whose cases may be suitable for the purpose, the symptoms of the diseases under which they may labour, and to lecture upon the nature of such diseases and the indications of cure and methods of treatment, which have by experience been found most successful in similar diseases.'

"In addition to the lectures on surgery, the professors of that branch will exhibit to their students, at stated periods, the cases of surgical diseases in the hospital of the Alms House; also the operations in surgery, which may occur in publick or private practice.

"Some other important practical advantages will be accessible to the students. These cannot be specified at present.

"The medical students or others will be allowed to attend either one, or more of the above named courses, as they may think proper. Those, who desire to obtain a medical degree, must attend two courses in each branch. The degrees will be conferred, as formerly, at the University.

"The professors possess a very valuable collection of anatomical preparations, which will greatly aid their demonstrations on recent subjects. They have a chemical apparatus, which is extensive and adequate to the performance of the experiments, which should illustrate lectures on chemistry. These are to be deposited in commodious apartments in a building, now preparing for the purposes of the institution. There is also an excellent library, established by the munificence of Ward Nicholas Boylston, Esq. the use of which will be enjoyed by the students.

"It is believed that the price of living in Boston will not greatly exceed that in country towns, at least to those who are willing to make some temporary sacrifices to the acquisition of knowledge, which will be permanently profitable to them. The professors will be able to point out suitable houses, where the students may be lodged and boarded. They will also endeavour to render the situation of the students comfortable, and to promote their improvement in medical learning, by every means in their power.

"Private pupils will be received by the professors on the usual terms.

"It is not thought necessary to state minutely the reasons, which have caused the establishment of this medical institution in the town of Boston, or to urge the superior advantages of a medical school, placed in a populous town. Boston contains from thirty to forty thousand inhabits, and is closely surrounded by the large towns of Charlestown, Cambridge, Ruxbury, and Dorchesser. The opportunity among so great a number of people, of observing the diseases and accidents incident to mankind, must be very extensive. The consequent collection of a large number of able practitioners, with whom the student may have an important literary intercourse, and the necessary concentration of medical knowledge, are advantages not to be overlooked. It is obvious that all these sources combined may afford to students a portion of useful information, in the course of three or four months, which they would in vain seek for in a long period of practice in the country.

The subscribers have founded an expectation on these circumstances that students will resort to Boston from every part of the country; that thus they shall be enabled to enlarge the institution in various ways, so that this shall become, what it ultimately should be, the Medical School of New England.

"We are Sir, with respect,
"Your very humble servants,
"JOHN WARREN,
"BENJAMIN WATERHOUSE,
"AARON DEXTER,
"JAMES JACKSON,
"JOHN C. WARREN,
"JOHN GORHAM."

---

*Case of Small Pox after Vaccination.*—Miss Reynholds, of King-street, Portman-square, now ten years of age, was vaccinated when three weeks old, by Mr. Griffith, of St. George's Hospital. Mr. Croft, of Burlington-street, and Mr. Forbes, of New-street, Hanover-square, with Mr. Griffith, pronounced the Vaccination to have regularly gone through its stages; and the cicatrix now remains in her arm, where the vaccine vesicle had been.

On Friday, August the 9th, 1811, Miss Reynholds was very unwell all the day, was feverish, and probably had been disordered the night before. She continued ill with the usual symptoms of fever till Sunday morning, when on getting out of bed eruptions were seen, which continued to increase all that day and following night. Dr. G. Pearson saw her for the first time (by desire of Mr. Forbes) on Tuesday. She was then much relieved of the fever, but had a considerable number of eruptions like the Small-pox. On Friday the 16th, when Dr. Pearson saw her again, the eruptions were ascertained, most unequivocally, to be those of Small-pox: most of them seemed to be already in a suppurated state, with an areola at their basis. On the face they were partially confluent. The preceding night, Thursday, she had been feverish, with quick pulse, and foul tongue; but without alarming symptoms.

On Tuesday, the 20th, the 12th day of the disease, and the 8th of the eruption, the pustules were scabbing, and many of them had already gone through that process on the face, but were still distinctly purulent on other parts. Notwithstanding the eruptions were very numerous, but little secondary fever had happened, and not much swelling of the face.

Saturday, the 24th, the 16th day of the disease, when Dr. Pearson visited the patient in company with Mr. Royston, most of the scabs had fallen off, leaving, as usual, distinct marks.

Miss Reynolds was seen by Dr. Domier, Mr. Croft, and Mr. Forbes, as well as the gentlemen before mentioned; and no doubt was entertained by any of them but that the disease was a case of genuine Small-pox.

---

*Case.*—A. S. a boy two years and eight months old, of a rachitic habit, was attacked with fever on the 24th of August, manifested by quick pulse, hot skin, and flushed face. There was no extraordinary restlessness, pain, or other indication of local affection as connected with the febrile state. On the 25th the action of the circulating system was considerably lessened, and at 12 o'clock his disease had so evidently decreased as to promise its speedy subsidence. At four in the afternoon his mother believed he suffered from pain in his bowels, which had not, however, been constipated during his indisposition, and he was unable to bear the erect position. Two hours after this, at six in the evening, enormous perspiration came on, especially from the scalp, and a night-cap was completely wetted in five minutes; the pulse was rapid, and had become feeble; the face pale and sunk; and the extremities cold. He vomited several times between seven and twelve, and at two in the morning of the 26th, expired.

An examination of the body by Mr. Brooks, the anatomist, took place 16 hours after death. The abdominal viscera was without inflammation, the intestines were in so fresh and perfect a state as to show, with great distinctness, the *glandula solitaria* and *agregata*, but in two places of that canal very complete introsusceptio had taken place without inflammation. Elsewhere there was no mark of disease. Did these introsusceptiones take place at four in the afternoon of the 25th, and is that derangement in the intestinal canal capable of producing the symptoms that followed, and so speedy a dissolution, without intervening inflammation? Is the sudden and excessive perspiration which here occurred, and particularly on the slalp, with rapid decrease of vital energy, indicatory of this peculiar derangement in the intestine?

---

*Lacerta agilis.*—In 1787, the common lizard was a popular medicine at Geneva for cutaneous complaints. The President of the Linnæan Society, who was then in that city, says that Dr. Butine, sen. a candid and well informed man, mentioned this circumstance to him. The muscular parts only of the animal were taken raw in a bolus, and proved violently sudorific. This remedy was, however, considered to hurt the lungs; occasioning coughs and spitting of blood.

---

Dr. Hosack, of New York, has begun to publish a periodical work, to be entitled the "American Medical Philosophical Register," from which much is expected. One of its principal objects will be to establish the line of distinction between those diseases which are of foreign growth, and such as are engendered at home: this will necessarily involve an investigation of the theories and opinions which have been disseminated on the yellow fever.

Process

*Process of making Sulphate of Magnesia, as employed at Lymington in Hampshire*.—At Lymington advantage is taken of the greater heat of the climate, to concentrate the sea water by spontaneous evaporation to about one sixth its bulk, before admitting it into the boilers. One kind of salt is chiefly prepared there, which most resembles in grain the stoved salt of Cheshire. The salt is not fished (as it is termed) out of the boiler, and drained in baskets; but the water is entirely evaporated, and the whole mass of salt taken out at once, every eight hours, and removed into troughs with holes in the bottom Through these it drains into pits made under ground, which receive the liquor called *bittern* or *bitter liquor*. Under the troughs, and in a line with the holes, are fixed upright stakes, on which a portion of salt that would otherwise have escaped crystallizes and forms, in the course of ten or twelve days, on each stake, a mass of sixty or eighty pounds. These lumps are called *salt-cats* They bear the proportion to the common salt, made from the same brine, of 1 ton to 100.

From the mother brine, or bitter liquor, which has drained into the pits, the sulphate of magnesia is made during the winter season, when the manufacture of salt is suspended, in consequence of the want of the temperature required, for the spontaneous evaporation of the sea-water. The process is a very simple one. The bitter liquor from the pits is boiled for some hours in the pans which are used in summer to prepare common salt; and the impurities which rise to the surface are removed by skimming. During the evaporation, a portion of common salt separates; and this, as it is too impure for use, is reserved for the purpose of concentrating the brine in summer. The evaporated bitter liquor is then removed into wooden coolers eight feet long, five feet wide, and one foot deep. In these it remains twenty-four hours, during which time, if the weather prove clear and cool, the sulphate of magnesia, or Epsom salt, crystallizes at the bottom of the coolers, in quantity equal to about one eighth of the boiled liquor. The uncrystallizable fluid is then let off through plug-holes at the bottom of the coolers; and the Epsom salt, after being drained in baskets, is deposited in the store-house. This is termed *single* Epsom salts, and after solution and a second crystallization, it acquires the name of *doub e* Epsom salts. Four or five tons of sulphate of magnesia are produced from a quantity of brine, which has yielded 100 tons of common, and one ton of cat salt.

---

*Case of Aneurism of the Aorta, which burst into the Pericardium.*—A robust man, 30 years of age, had been afflicted for five months with an extreme difficulty of respiration. He had a slight cough and a constant expectoration of thin frothy mucus. His pulse was frequent, but did not intermit. No increase during this time was observed in the symptoms, when after eating a very hearty meal, he gave a deep groan and instantly expired. Upon dissection, a large aneurism wss found to occupy the whole of the arch, and a great part of the ascending aorta, beginning about half an inch above the semi-lunar valves. The sac extended in every direction, but more particularly downwards, and its origin above the valves was bounded by the pericardium, into which it had burst by an aperture large enough to admit three fingers. The large brahches of the arch went off from the superior part of the sac, which which contained about a pint of clotted blood, and a considerable quantity of lamellated coagulum. The trachea adhered firmly to the sac, and the pericardium was full of blood.—*Lond. Med. Rev.*

---

*Poison of the Upas.*—This vegetable poison, whether introduced into the system by the blood-vessels or the lymphatics, by the intestines or by wounds, was equally fatal; the animals dying universally convulsed. From experiments made by Messrs. Majendie and Delisle, it appeared particularly to affect the spinal marrow, and to enter the system only by means of the circulation. It seems to act very indirectly on the brain, and shews an independence between it and the spinal marrow, not indicated by anatomy.

---

Messrs. Vauqelin and Sage have found that juice of belladonna, when swallowed by animals, produces in them a delirium similar to that occasioned by opium.

---

The effect of different gasses injected into the blood-vessels has been examined by Dr. Nysten, of the French Institute. Atmospheric air, oxygen, nitrous oxide, carbonic oxide, sulphuretted hydrogen, nitric oxide, and nitrogen, were not deleterious. Oximuriatic, ammoniacal and nitrous acid gasses appeared to act by violently irritating the right auricle and pulmonary ventricle. Sulphuretted hydrogen, nitric oxide, and nitrogen, were injurious to the contractability of these parts. Some others so changed the blood, that respiration was unable to convert it from venous to arterial.

---

For the sting of the weever (Trachinus draco Linn.) and against the effects of the poison of the tarantula, M. Sage on a paper expressly on the subject, recommends the internal and external use of the volatile alkali.

---

THEATRE OF ANATOMY, Blenheim-street, Great Marlborough-street.—The Autumnal Course of Lectures on Anatomy, Physiology, and Surgery, will be commenced by Mr. BROOKES on Tuesday, the 1st of October, at Two o'clock.

Anatomical Converzationes will be held weekly, when the different subjects treated of will be discussed familiarly, and the Students' views forwarded.—To these none but Pupils can be admitted.

Spacious apartments, thoroughly ventilated, and replete with every convenience, are open all the morning for the purposes of dissecting and injecting, where Mr. Brookes attends to direct the Students, and demonstrate the various parts as they appear on Dissection.

The inconveniences usually attending Anatomical Investigations, are counteracted by an antiseptic process. Pupils may be accommodated in the House. Gentlemen established in Practice, desirous of renewing their Anatomical Knowledge, may be accommodated wtih an Apartment to dissect in privately.

THEATRE

Theatre of Anatomy, Great Windmill-street.—The following Lectures, given in this School, commence in October, and terminate in May: the hours of delivering them are so arranged as not to interfere with each other, or at the time of attendance at the hospital.

Chemistry, every morning, from eight until nine o'clock; by William T. Brande, F.R.S.

Materia Medica, twice in the week, from nine until ten o'clock in the morning; by William T. Brande, F. R. S.

Theory and Practice of Physic, by John Cooke, M. D. F. A. S. and Philip Roget, M.D., and late Physician to the London Hospital; from nine until ten o'clock, four mornings in the week.

Anatomy, Physiology, Pathology, and Surgery, by James Wilson, F. R. S.; and B. C. Brodie, F. R. S.; from two till four o'clock in the afternoon as usual. The Anatomical Lectures commence on the first of October, at two o'clock.

Surgery, by B. C. Brodie, F. R. S. Assistant-Surgeon to St. George's Hospital, from seven until eight, three evenings in the week.

A room for Practical Anatomy is open every morning, from eight o'clock until two in the afternoon, under the direction of Mr. Wilson, and Mr. Brodie; where a regular demonstration of the dissected parts is given daily, from one until two o'clock.

Twelve Gratuitous Lectures, on the principal Operations of Surgery, are given to the Pupils of St. George's Hospital during the Season, by Everard Home, Esq. F. R. S. Serjeant-Surgeon to his Majesty, and Surgeon to St. George's Hospital.

---

St. Thomas's and Guy's Hospitals.—The winter Courses of Lectures at these adjoining Hospitals will commence the first week of October; viz.

*At St. Thomas's.*—Anatomy and the Operations of Surgery, by Mr. Cline, and Mr. Astley Cooper.

The Principals and Practice of Surgery, by Mr. A. Cooper.

*At Guy's.*—Practice of Medicine, by Dr. Babington and Dr. Curry.

Chemistry, by Dr. Babington, Dr. Marcet, and Mr. Allen.

Experimental Philosophy, by Mr. Allen.

Theory of Medicine, and Materia Medica, by Dr. Curry and Dr. Cholmeley.

Midwifery and Diseases of Women and Children, by Dr. Haighton.

Physiology, or Laws of the Animal Œconomy, by Dr. Haighton.

Structure and Diseases of the Teeth, by Mr. Fox.

These several Lectures are so arranged, that no two of them interfere in the hours of attendance; and the whole is calculated to form a Complete Course of Medical and Chirurgical Instructions.

Mr.

Mr. Armiger, Surgeon Extraordinary to his Royal Highness the Duke of Kent, and Surgeon to the Eastern Dispensary, will commence a Course of Lectures on the Operations of Surgery, on Monday, September 2d, at three o'clock.

These Lectures, of which it is the principal object to demonstrate the manner of performing the operations, will be continued every day at the same hour till the end of the month.

On Tuesday, the first of October, Mr. A. will commence his Autumn Course of Anatomy and Surgery.

---

Mr. Stevenson will commence his Autumnal Course of Lectures on the Anatomy, Physiology, and Diseases of the Eye and Ear, on Tuesday, October 22d, at Seven o'clock in the evening, at his house, Great Russel Street, Bloomsbury Square.

---

Anatomical Theatre, Bristol.—Mr. T. Shute will commence his Winter Course of Lectures on Anatomy, Physiology, and the Principles of and Operations in Surgery, on Tuesday, October the first, at eight o'clock in the morning.

---

London Hospital.—Dr. Buxton's Autumnal course of Lectures on the Practice of Medicine, will be commenced on Wednesday, October 2d.

---

Dr. Clutterbuck will begin his Autumn course of Lectures on the Theory and Practice of Physic, Materia Medica, and Chemistry, on Monday, Oct. 7, at Ten o'clock in the morning, at his house, New Bridge Street, where they will be continued daily at the same hour.: viz. Theory and Practice, on Mondays, Wednesdays, and Fridays; Materia Medica and Chemistry, on Tuesdays, Thursdays, and Saturdays.

Clinical Lectures will be given occasionally during the Winter, on the most remarkable cases that occur in the practice of the General Dispensary. Pupils preparing to pass the Medical Boards will be admitted to private Examinations.

---

Dr. Reid's next course of Lectures on the Theory and Practice of Medicine, will commence, at nine o'clock in the morning, on Monday, the Fourteenth of October, at Dr. Reid's house, Grenville Street, Brunswick-square.

---

Dr. Tuthill's Lectures on the Practice of Physic, and on the Laws and Operations of Chemistry, will commence on Monday, the 7th of October. For particulars *vid. Cover of the Journal.*

---

Dr. Adams's course of Lectures on the Institutes and Paactice of Medicine, will commence about the beginning of October, at his house, Hatton Garden. Syllabus and Prospectus to be had of the different Medical Booksellers, and at Dr. Adams's residence.

Medical

Medical and Chemical Lectures, George-street, Hanover-square, and St. George's Hospital.—The Medical Lectures will recommence as usual, the first week of October, at eight in the morning, and the Chemical at a quarter after nine o'clock; by George Pearson, M. D. F. R. S. Physician in Ordinary to their Royal Highnesses the Duke and Duchess of York, and their Household; Senior Physician of St. George's Hospital; of the College of Physicians; Fellow of the Imperial Medico-Chirurgical Academy of St. Petersburgh, of the College of Physicians, &c. &c. &c.

Clinical Lectures are given on the patients registered in St. George's Hospital, every Saturday morning at nine o'clock.

---

Lectures on Midwifery, Theoretical and Practical, including the Diseases of Women and Infants as connected therewith, will commence on Monday morning, the 14th of October, at half past ten; and on Tuesday evening, the 15th, at seven; by Dr. Clough, Physician Accoucheur to the St. Marylebone General Dispensary, &c. at his Lecture room, 68, Berners' Street, where a Syllabus and Prospectus may be had.

---

Dr. Clarke, and Mr. Clarke, will begin the Winter course of their Lectures on Midwifery, and the Diseases of Women and Children, on Friday, October 4th.

The Lectures are read every day at the house of Mr. Clarke, Upper John Street, Golden-square, from a quarter past ten o'clock in the morning till a quarter past eleven, for the convenience of Students attending the hospitals.

The Students will be provided with cases when qualified to attend them.

---

Mr. Moor, Surgeon-Dentist to her Royal Highness the Duchess of York, will commence a course of Lectures on the Structure and Diseases of the Teeth, on the tenth of October; in which will be explained the complete practice of a Dentist.

---

Dr. Ramsbotham will commence his Lectures on the Science and Practice of Midwifery, on Monday October 7, at his house in the Old Jewry.

A Gentleman may be accommodated as house pupil.

---

Dr. Thynne will commence his course of Lectures on Midwifery, on Tuesday, October the first, at the Theatre, Bartholomew's Hospital, and in Berners' Street.

---

Theatre, London Hospital.—Dr. Dennison, and Dr. Byam Dennison, will commence their first course of Lectures on the Theory and Practice of Midwifery, and the Diseases of Women and Children, on Tuesday, October 8th, at eleven o'clock.

